# Biomimetic Cell Membrane-Based Drug Delivery Systems for Oral Diseases: Engineering Strategies, Targeting Mechanisms, and Translational Challenges

**DOI:** 10.3390/pharmaceutics18070799

**Published:** 2026-06-29

**Authors:** Zeyuan Xie, Lingling Zhang, Chengcheng Yin, Xu Zhang, Yanqin Lu

**Affiliations:** 1 Department of Orthodontics, Xiangya Stomatological Hospital and Xiangya School of Stomatology, Central South University, Changsha 410008, China; 8302210108@csu.edu.cn (Z.X.); doczll@csu.edu.cn (L.Z.); 2 Hunan Engineering Research Center for Oral Digital Intelligence and Personalized Medicine, Changsha 410008, China; 3 Hunan Key Laboratory of Oral Health Research, Changsha 410008, China; 4 Department of Dermatology, National Engineering Research Center of Personalized Diagnostic and Therapeutic Technology, Xiangya Hospital, Central South University, Changsha 410008, China; sunshine109123@csu.edu.cn; 5 FuRong Laboratory, Changsha 410008, China; 6 Hospital of Stomatology, Jilin University, Changchun 130021, China; yinchengcheng@jlu.edu.cn; 7 Jilin Provincial Key Laboratory of Oral and Craniofacial Diseases & Tissue Reconstruction, Changchun 130021, China

**Keywords:** biomimetic drug delivery, cell membrane-coated nanoparticles, targeted delivery, oral diseases, nanomedicine

## Abstract

Oral diseases, encompassing conditions such as periodontitis, head and neck squamous cell carcinoma, pulpitis, and mucosal infections, remain a major global health burden due to their high prevalence and complex, multifactorial pathophysiology. The unique anatomical structure of the oral cavity, together with persistent microbial challenges and dynamic immune responses, imposes substantial limitations on conventional drug delivery strategies. Biomimetic cell membrane-based materials have recently emerged as a promising class of delivery platforms, leveraging natural biological interfaces to confer inherent biocompatibility, immune evasion, prolonged circulation, specific targeting, and biofilm-interactive capabilities. These features position them as a transformative approach for improving therapeutic precision and efficacy in oral disease management. In this review, we provide a systematic and materials-oriented overview of biomimetic cell membrane-based drug delivery systems. Specifically, we discuss: (1) the biological sources, classification, and physicochemical properties of membrane-coated systems, along with their fabrication and engineering strategies; (2) the mechanistic basis of targeting, immune modulation, and nanobiointerface interactions, and their applications across representative oral diseases; and (3) current challenges, including scalable manufacturing, functional controllability, biosafety, and clinical translation. Furthermore, we highlight emerging directions such as stimuli-responsive membrane systems and multifunctional integrated platforms, aiming to provide a conceptual framework for the rational design and clinical advancement of biomimetic drug delivery systems in complex disease settings.

## 1. Introduction

The oral cavity constitutes a uniquely complex physiological environment that hosts a diverse spectrum of diseases, necessitating multifaceted therapeutic strategies. Despite advances in clinical management, the global burden of oral diseases remains substantial, encompassing malignancies, chronic inflammatory conditions, and infectious disorders. Head and neck squamous cell carcinoma (HNSCC) remains a leading cause of cancer-related mortality, with nearly 390,000 new cases of lip and oral cavity cancer reported annually [1] and a 5-year survival rate of approximately 50% [2]. In parallel, chronic inflammatory diseases impose a significant impact on public health; severe periodontitis affects roughly 19% in people over 15 years old [3] and is a major risk factor for systemic comorbidities such as diabetes [4]. Furthermore, infectious diseases of dental tissues are ubiquitous, with untreated dental caries in permanent teeth that affects approximately 2 billion individuals worldwide, often progressing to debilitating pulpitis and periapical lesions [3]. Similarly, oral mucosal disorders, including recurrent aphthous stomatitis and lichen planus, impact up to 25% of the population [5].

The clinical management of these conditions is fundamentally constrained by the limitations of conventional drug delivery systems (DDSs) and the intrinsic barriers of the oral environment. In HNSCC, standard treatments such as surgery, radiotherapy, and chemotherapy suffer from limited specificity [6], unwanted side effects [7], and therapeutic resistance [8]. For diseases requiring local administration, continuous salivary secretion (0.5 to 1.5 mL/min), mechanical friction, and extensive vascularization of the oral mucosa result in rapid drug clearance, significantly reducing retention time and bioavailability [9]. Furthermore, the stratified squamous epithelium acts as a dense physical barrier restricting drug penetration [10], while complex microbial biofilms further impede drug diffusion through their protective extracellular matrix [11].

**Figure 1:**
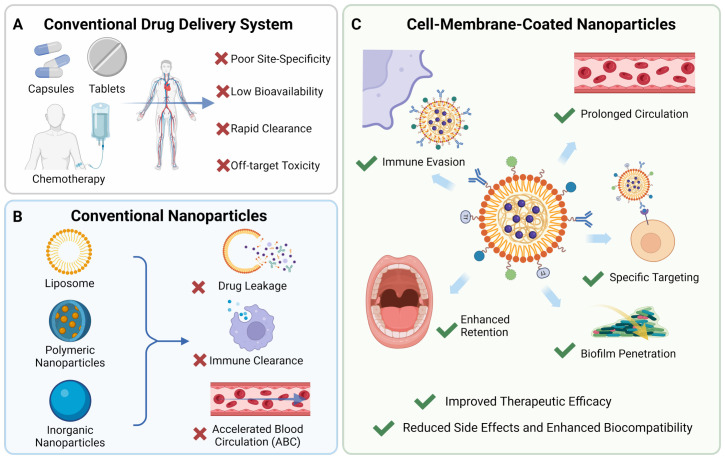
Limitations of traditional drug delivery systems and advantages of cell membrane-coated nanoparticles: (**A**) limitations of capsules, tablets and traditional chemotherapy, (**B**) limitations of traditional nanoparticles and (**C**) advantages of CMCNPs. (Created in BioRender. Lin, Z. (2026). https://BioRender.com/lfikcdc, accessed on 26 May 2026).

Conventional DDS, including tablets, capsules, and basic formulations, as well as many early nanotechnology-based systems (e.g., liposomes, polymeric nanoparticles, and inorganic carriers), often fail to adequately address these challenges. They typically exhibit poor site-specific accumulation, unfavorable biodistribution, rapid immune clearance [12] or accelerated blood clearance (ABC phenomenon) [13], premature drug leakage, suboptimal controlled release profiles [14], limited mucus penetration or biofilm disruption, and insufficient targeting in the dynamic oral microenvironment, ultimately resulting in suboptimal therapeutic efficacy and potential off-target toxicity [15] (Figure 1A,B).

To overcome these barriers, biomimetic strategies have emerged as a promising paradigm for next-generation drug delivery. Among them, cell membrane-coated nanoparticles (CMCNPs) leverage the intrinsic biological functions of natural membranes (e.g., from red blood cells, platelets, immune cells, or cancer cells) to achieve superior immune evasion via surface markers like CD47, prolonged circulation (often outperforming PEGylation), enhanced bioavailability, homotypic or specific targeting, biofilm penetration, and better biocompatibility compared to conventional synthetic nanocarriers [16]. These platforms inherit natural cell-like properties, enabling them to evade macrophage uptake more effectively, interact dynamically with the oral microenvironment (including immune modulation and pathogen decoying), and provide multifunctional advantages such as extended retention and precise delivery that conventional nanocarriers struggle to achieve [17].

The integration of biomimetic cell membrane engineering with oral disease therapeutics represents a novel advancement, as it specifically tailors these platforms to the unique challenges of the oral cavity—such as salivary clearance, mucosal barriers, and polymicrobial biofilms—where traditional nanocarriers have shown limited translational success. Recent applications demonstrate their potential in HNSCC (e.g., targeted chemotherapy and phototherapy), periodontitis (antimicrobial and immunomodulatory effects), and other oral conditions, offering improved efficacy, reduced side effects, and better translational potential compared to conventional approaches [18] (Figure 1C).

In this review, we provide a comprehensive and systematic overview of biomimetic cell membrane-based materials for oral disease treatment, with a focus on their design principles, functional mechanisms, and translational prospects.

## 2. Fundamental Theories and Technologies

This section systematically elaborates on the basic principles of these materials, from their diverse biological origins to the intricate technologies employed for their preparation and engineering modification, focusing on their adaptability and performance within the unique physiological constraints of the oral environment.

### 2.1. Membrane Sources

Biomimetic cell membrane materials are derived from various biological cells, each cell type containing unique surface proteins and lipids. These components confer corresponding functionalities to the resulting nanocarriers. The selection of the membrane source is a critical design parameter, as it fundamentally determines immune evasion capabilities, targeting specificity, and multiple biological interactions [19,20,21].

#### 2.1.1. Erythrocyte (Red Blood Cell—RBC) Membrane Materials

RBC membranes serve as a foundational platform in cell membrane-coated nanotechnology and remain among the most extensively utilized sources for biomimetic coatings due to their high natural abundance, straightforward isolation protocols, and exceptional biocompatibility [22,23,24]. RBCs possess a uniquely simplified biological structure—devoid of a nucleus, organelles, and mitochondria—which effectively minimizes metabolic activity and inherent immunogenicity, contributing to an impressive half-life of systemic circulation which up to several weeks [25,26]. Their membranes exhibit specialized mechanical and biochemical properties that ensure both structural stability and robust immune evasion. Specifically, the spectrin–actin cytoskeleton, anchored by ankyrin and band-3 complexes, imparts remarkable elasticity and mechanical resilience, allowing RBCs to undergo significant deformation when traversing narrow capillaries without fragmentation [27,28].

Furthermore, the RBC lipid bilayer maintains a distinct phospholipid asymmetry: phosphatidylserine (PS) and phosphatidylethanolamine (PE) are sequestered in the inner leaflet, while phosphatidylcholine (PC) and sphingomyelin (SM) are enriched in the outer leaflet [29]. This intricate organization maintains membrane fluidity and prevents detrimental cell aggregation. Concurrently, negatively charged sialylated glycoproteins on the surface reduce non-specific protein adsorption and complement activation [30]. Crucial surface proteins, including CD47, CD55, and CD59, further protect these cells from phagocytosis and complement-mediated lysis [31]. The “stealth” capability is primarily driven by CD47, which acts as a “don’t eat me” signal by interacting with signal regulatory protein alpha (SIRPα) on macrophages to actively inhibit phagocytosis, thereby prolonging the systemic circulation of RBC membrane-coated nanoparticles (RBCM-NPs) [32]. Mechanistically, the interaction between CD47 and SIRPα initiates phosphorylation of immunoreceptor tyrosine-based inhibitory motifs (ITIMs) within the cytoplasmic domain of SIRPα, leading to the recruitment of SHP-1 and SHP-2 phosphatases and subsequent inhibition of macrophage cytoskeletal rearrangement and phagocytic synapse formation [33,34]. In addition, RBC membrane camouflage may circumvent the ABC phenomenon associated with repeated administration of PEGylated nanocarriers, thereby offering superior long-term biocompatibility and immune tolerance [35].

When coating nanoparticles, RBC membranes significantly prolong circulation time and mitigate clearance by the mononuclear phagocyte system (MPS) [24,36]. This character is highly advantageous for targeted drug delivery, facilitating enhanced accumulation at pathological sites such as malignant tumors or inflamed tissues [37,38]. RBC membranes can be efficiently harvested in large quantities through hypotonic lysis and purified with high yields, rendering them highly suitable for scalable pharmaceutical production [24].

#### 2.1.2. Platelet Membrane Materials

Platelets, which recognize activated endothelium and exposed extracellular matrix at sites of tissue injury [39,40], are crucial mediators in hemostasis, thrombosis, and tissue regeneration. They serve as primary responders to vascular injury and inflammatory stimuli through their natural affinity for damaged tissues, specific pathogens, and malignant cells [41,42]. Using these innate homing capabilities, platelet membrane-coated nanoparticles (PMCNPs) can effectively target tumor vasculature [43,44], metastatic lesions [43], inflamed tissue/environment [45], such as the site of periodontitis [46], and sites of oral surgical intervention [46]. Their surface repertoire of receptors—including integrins, P-selectin, GPIIb/IIIa, and CD41—facilitates site-specific adhesion to activated endothelial cells and tumor cells, thereby enhancing drug accumulation at pathological sites while mitigating systemic off-target toxicity [23,25,45,47].

Similar to erythrocyte membranes, platelet membranes retain immune-regulatory surface proteins, particularly CD47, which suppresses macrophage-mediated phagocytosis via interaction with signal regulatory protein alpha (SIRPα) [48]. Therefore, PMCNPs exhibit immune evasion properties comparable to red blood cells, ensuring prolonged systemic circulation [47]. Also, platelet membranes can specifically bind to oral pathogens such as *Porphyromonas gingivalis* [45] and *Staphylococcus aureus* [49], through Toll-like receptors (TLRs) and microbicidal peptides [50], enabling the precise delivery of concentrated antimicrobial agents to infectious foci. Their inherent role in modulating wound repair further underscores their potential in the treatment of periodontitis and peri-implantitis [45].

#### 2.1.3. Leukocyte (White Blood Cell—WBC)-Derived Membrane Materials

WBCs, encompassing neutrophils, macrophages, dendritic cells, T cells, and natural killer (NK) cells, are biologically programmed to navigate toward sites of inflammation, infection, and malignancy via sophisticated migratory and adhesive mechanisms [51]. Coating nanocarriers with WBC-derived membranes (WBCM-NPs) harnesses these chemotactic capabilities for targeted therapy [52]. Macrophage-derived membranes are particularly advantageous for managing inflammatory oral conditions, such as periodontitis, as they express a high density of pro-inflammatory cytokine receptors, including tumor necrosis factor-alpha receptor (TNF-αR) and interleukin-6 receptor (IL-6R) [53]. These membranes function as “nanodecoys” or “molecular sponges” that competitively bind and neutralize inflammatory mediators (e.g., TNF-α, IL-1, and IL-6) released during host immune responses to periodontal pathogens [54,55]. Studies have demonstrated that macrophage membrane-functionalized systems can induce a phenotypic shift in local macrophage populations from a pro-inflammatory M1 state to a pro-healing M2 phenotype, thus restoring the balance of inflammation–osteogenesis coupling and promoting alveolar bone regeneration in vivo [46,56]. Similarly, neutrophil membrane-coated nanoparticles recapitulate the natural leukocyte adhesion cascade by leveraging key surface receptors, specifically $\beta_{2}$ integrins (e.g., LFA-1 and Mac-1) and selectin ligands (e.g., PSGL-1) [57,58]. These proteins mediate specific molecular recognition of ICAM-1 and P/E-selectins upregulated on inflamed endothelium, thereby providing a robust mechanism for targeted anti-inflammatory drug delivery [59]. Dendritic cell membranes, utilized for their potent antigen-presenting capacity and CCR7 expression, facilitate targeted delivery to the lymphatic system—a critical pathway for intercepting oral squamous cell carcinoma metastasis [60,61]. CCR7 responds primarily to the chemokines CCL19 and CCL21, among which CCL21 is constitutively expressed by lymphatic endothelial cells and establishes chemotactic gradients that guide dendritic cell migration toward draining lymph nodes [62,63,64]. This feature is particularly valuable for targeting metastatic dissemination through cervical lymphatic networks in oral squamous cell carcinoma. Furthermore, T cell and NK cell membranes enhance oncological immunotherapy through specialized immune modulation [65,66]. These biomimetic platforms carry essential adhesion molecules (e.g., LFA-1, CD45) that ensure precision guidance to activated endothelium and specific diseased cell types [67,68].

#### 2.1.4. Cancer Cell-Derived Membrane Materials

Cancer cell membranes (CCMs) are distinguished by their innate “homologous targeting” capability, which enables functionalized nanoparticles (NPs) to preferentially adhere to and be internalized by parent cancer cells or those of an identical histological lineage [69,70]. This homotypic recognition is primarily mediated by a dense repertoire of overexpressed surface adhesion molecules and receptors, such as CD44, E-cadherin, and galectin-3, which facilitate stable interfacial interactions [71]. By implementing this “cancer-catch-cancer” strategy, CCM-coated platforms—particularly those derived from oral squamous cell carcinoma (OSCC)—significantly enhance drug accumulation at the primary tumor site while simultaneously suppressing distant metastasis [72]. Beyond localized targeting, CCMs retain a comprehensive spectrum of tumor-associated antigens (TAAs), acting as potent nanovaccines that stimulate the host’s immune system for precision immunotherapy [73,74,75]. For instance, CCM-functionalized Co-fc nanoparticles have demonstrated efficacy in targeting OSCC through the inhibition of autophagy pathways [73]. Furthermore, these biomimetic shells allow nanocarriers to evade immune surveillance and penetrate intricate biological barriers, thereby circumventing nucleophile-mediated resistance in cisplatin delivery [76,77,78]. These advantages are attributed to enhanced compatibility with the tumor microenvironment (TME), facilitating deeper penetration into dense tumor tissues [79]. In comparison with conventional synthetic nanocarriers, CCM-coated systems exhibit reduced nonspecific clearance and superior intratumoral retention due to their endogenous membrane composition and tumor-mimicking surface properties.

#### 2.1.5. Stem Cell-Derived Membrane Materials

Stem cell membranes serve as a versatile platform in cell membrane-coated nanotechnology and are increasingly utilized for biomimetic coatings due to their exceptional biocompatibility, intrinsic tissue-specific homing properties, and immunomodulatory capabilities [80,81,82]. Stem cells, particularly mesenchymal stem cells (MSCs), possess multipotency and self-renewal abilities—devoid of the rigid constraints seen in differentiated cells—which minimize immunogenicity and enable seamless integration into host tissues, contributing to prolonged systemic circulation [83]. Their membranes exhibit specialized biochemical properties that ensure both structural integrity and effective immune evasion. Specifically, the phospholipid bilayer maintains asymmetry, with the exoplasmic leaflet enriched in PC and SM, while the cytoplasmic leaflet is composed largely of PE, PS, and phosphatidylinositol (PI); additionally, there is a marked asymmetry in lipid unsaturation, with the cytoplasmic leaflet being approximately twice as unsaturated as the exoplasmic leaflet, supporting membrane fluidity, signaling, and preventing unwanted aggregation [84,85].

Furthermore, stem cell membranes are adorned with a repertoire of surface receptors and glycoproteins, such as CXCR4, CD44, CD147, and CD138, which facilitate chemotactic migration and adhesion to inflamed or tumorous sites [86]. The homing capability is primarily driven by interactions with ligands like SDF-1 (via CXCR4), PDGF (via PDGFR), and VEGF (via VEGFR), acting as “find me” signals that guide the cells to pathological microenvironments, thereby enhancing targeted accumulation while actively suppressing immune recognition through low MHC expression [87].

When coating nanoparticles, stem cell membranes significantly prolong circulation time and mitigate clearance by the MPS [88]. This extended circulation profile is highly advantageous for targeted drug delivery, facilitating enhanced accumulation at pathological sites such as tumors or inflamed tissues [89]. Stem cell membranes can be efficiently harvested from sources like bone marrow, adipose tissue, or umbilical cords through hypotonic lysis, ultracentrifugation, or extrusion, rendering them suitable for scalable biomedical production [24].

#### 2.1.6. Bacterial Membrane Materials

Bacterial outer membranes (BOMs) and outer membrane vesicles (OMVs)—nanoscale structures ranging from 20 to 300 nm—serve as an innovative platform in cell membrane-coated nanotechnology, increasingly utilized for biomimetic coatings due to their natural secretion by Gram-negative bacteria, high immunogenicity, and ability to carry virulence factors for targeted antimicrobial and immunotherapeutic applications [90]. Their membranes exhibit specialized mechanical and biochemical properties that ensure structural integrity and potent biological activity. Specifically, the phospholipid bilayer maintains asymmetry, with the inner leaflet composed primarily of PE, phosphatidylglycerol (PG), and cardiolipin (CL), while the outer leaflet is dominated by lipopolysaccharides (LPSs), which includes lipid A (a phosphorylated glucosamine disaccharide with multiple fatty acid chains), core oligosaccharides (containing sugars like heptose and 3-deoxy-D-manno-octulosonic acid), and O-antigen chains (repeating polysaccharide units); this organization supports membrane stability, prevents unwanted aggregation, and facilitates interactions with host immune systems through Toll-like receptor activation [91].

Furthermore, bacterial membranes are enriched with surface proteins and virulence factors, such as porins (e.g., OmpA, OmpC), adhesins, proteases, and toxins, which enable adhesion to host tissues and enzymatic degradation of extracellular matrices [92]. The immunogenic capability is primarily driven by LPS and flagellin components, acting as pathogen-associated molecular patterns (PAMPs) that stimulate innate immune responses via pattern recognition receptors like TLR4 and TLR5, thereby enhancing targeted accumulation in infected sites while promoting cytokine release and macrophage activation to combat bacterial infections [93].

When coating nanoparticles, bacterial membranes significantly enhance biofilm penetration and mitigate antimicrobial resistance by facilitating the delivery of antibiotics into polymicrobial communities, such as those in dental caries and periodontal diseases [94]. This biofilm-disrupting profile is highly advantageous for targeted drug delivery, enabling enhanced accumulation at pathological sites such as chronic wounds or tumor microenvironments through “pathogen-targeting” mechanisms [95]. Additionally, hybrid vesicles—integrating BOMs with CCMs—have been developed as “Trojan Horse” systems for synergistic chemo-immunotherapy, combining the tumor-homing precision of CCMs with the adjuvant effects of bacterial LPS to elicit robust antitumor immune responses [96]. Bacterial membranes can be efficiently harvested in large quantities from culture supernatants through hypotonic lysis, filtration (e.g., 0.45 μm to remove cells), ultrafiltration (e.g., 100 kDa cutoff), and ultracentrifugation (e.g., 100,000× *g*), rendering them highly suitable for scalable pharmaceutical production [97].

Despite their promising biomedical applications, bacterial membrane-derived systems also present important biosafety concerns. LPS, particularly its lipid A component, may induce excessive inflammatory responses and systemic toxicity if not carefully controlled [98,99]. In addition, variability in bacterial strains, heterogeneity of vesicular cargo, and maintenance of batch-to-batch consistency remain major challenges for clinical translation. Consequently, strategies such as detoxified lipid A engineering and genetically modified low-endotoxin OMVs should be actively explored to improve safety profiles.

The core characteristics of diverse biomimetic membrane coatings are collated in Table 1.

Although diverse cell membranes show great potential in biomimetic nanomedicine, no single source satisfies all requirements for immune evasion, targeting specificity, and biosafety. We believe the major future direction for membrane-coated nanodelivery systems will center on hybridized membrane systems to integrate the distinct intrinsic properties of multiple cell sources. For instance, fusing erythrocyte membranes (prolonged circulation) with leukocyte, platelet (inflammatory targeting), or cancer cell membranes (homologous tumor recognition) can yield next-generation nanocarriers with synergistically enhanced performance.

Additionally, since therapeutic efficacy is inherently governed by the native membrane origin and its bioactive composition, several translational challenges regarding these biological sources must be addressed. The long-term biosafety of tumor- or bacterial-derived membranes, alongside the immunogenic heterogeneity among donors or cell lines, requires systematic investigation. Future research should therefore prioritize long-term biosafety assessments of these diverse cell sources to facilitate clinical translation.

### 2.2. Functional Optimization and Modification Technologies

Modern biomimetics transcends simple replication by employing engineering techniques to enhance cell membrane functions. It is primarily categorized into physical modification techniques, chemical modification techniques, and genetic engineering modification techniques.

#### 2.2.1. Physical Modification Techniques

- Lipid Insertion: Exogenous molecules tethered to lipid anchors—typically 1,2-distearoyl-sn-glycero-3-phosphoethanolamine-poly(ethylene glycol) (DSPE-PEG)—can be spontaneously incorporated into the membrane bilayer via hydrophobic interactions during co-incubation [100]. This non-destructive method allows for the decoration of membranes with targeting ligands (e.g., folate, aptamers) or imaging probes while preserving the topology and functionality of native transmembrane proteins, as the process avoids harsh chemical reagents [101].
- Membrane Hybridization:Membrane hybridization refers to the physical fusion of membranes derived from two or more distinct cell types, typically achieved by extrusion or sonication, to construct multifunctional chimeric vesicles that integrate the biological properties of each membrane source [102]. This strategy enables the simultaneous combination of complementary functionalities, such as immune modulation, prolonged circulation, and homologous targeting, within a single biomimetic nanoplatform. A representative example is the “DREAM” nanoplatform reported by Li et al., which was fabricated by fusing M1 macrophage membranes with oral squamous cell carcinoma (OSCC) cell membranes [103]. By integrating immune-activating macrophage components with tumor cell membrane-mediated homologous recognition, this hybrid system effectively modulated the tumor microenvironment through the regulation of glycolytic metabolism and the induction of cuproptosis, thereby enhancing antitumor immunotherapeutic efficacy in vivo.

#### 2.2.2. Chemical Modification Techniques

- Ligand Conjugation and Functional Group Coupling: To endow biomimetic nanocarriers with active targeting capability, functional ligands such as antibodies (e.g., anti-PD-L1) [104], peptides (e.g., cRGD) [105], or small molecules can be covalently conjugated to membrane surface proteins through well-established bioconjugation chemistries. A widely adopted strategy employs heterobifunctional linkers, such as NHS–PEG–maleimide, in which the N-hydroxysuccinimide (NHS) ester reacts with primary amine groups (−NH_2_) on the lysine residues of membrane proteins to form stable amide bonds, while the maleimide moiety enables subsequent conjugation with thiol-containing ligands [106]. This approach allows versatile ligand grafting while maintaining membrane integrity. Alternatively, metabolic engineering offers a bioorthogonal route for membrane functionalization by introducing azide groups onto cell-surface glycans through the incorporation of unnatural monosaccharide analogues. The azide-modified membranes can then undergo copper-free click chemistry, such as strain-promoted alkyne–azide cycloaddition (SPAAC), with dibenzocyclooctyne (DBCO)-functionalized ligands under physiological conditions [107]. Owing to its high specificity, rapid kinetics, and catalyst-free nature, this bioorthogonal strategy enables efficient surface modification without disrupting native membrane protein structure or biological function.
- Stimuli-Responsive Modifications: To achieve spatiotemporally controlled drug release, stimuli-responsive linkers can be incorporated into the biomimetic membrane shell. Reactive oxygen species (ROS)-responsive thioketal linkers have been widely employed to conjugate therapeutic cargos, which undergo oxidative cleavage in ROS-enriched pathological microenvironments such as tumors or inflamed tissues, thereby triggering on-demand payload release [108]. In addition, reduction-responsive disulfide bonds are frequently introduced into membrane-associated conjugates to enable intracellular drug release. These linkages remain stable in the extracellular milieu but are rapidly cleaved in the glutathione-rich cytosolic environment following cellular internalization, providing an effective mechanism for selective intracellular payload liberation [109].

#### 2.2.3. Genetic Modification Techniques

Genetic modification enables the reprogramming of source cells prior to membrane isolation, allowing the de novo expression of functional membrane proteins or receptors with correct folding and preserved bioactivity. This strategy is typically achieved through viral transduction or gene-editing techniques, followed by selection to establish stable cell lines with robust surface expression of the desired proteins. The engineered cell membranes can subsequently be harvested and used to coat nanoparticles, thereby transferring genetically encoded targeting or immunomodulatory functions to the resulting biomimetic nanocarriers [110].

- Macrophage Membrane Engineering: Toll-like receptor 4 (TLR4) is a pivotal pattern-recognition receptor responsible for sensing LPS from Gram-negative bacteria. Genetic overexpression of TLR4 in macrophages prior to membrane extraction enables the fabrication of TLR4-enriched macrophage membrane-coated nanoparticles that function as high-affinity decoys for bacterial endotoxins. In a representative study, macrophages were genetically engineered to overexpress TLR4, and the resulting membranes were employed to construct biomimetic nanoplatforms. These TLR4-rich systems effectively captured bacteria and neutralized LPS in vitro, suppressed NF-κB-mediated inflammatory signaling, and significantly alleviated inflammation-induced alveolar bone loss in a ligature-induced periodontitis model [46].
- Fibroblast Membrane Engineering: Chemokine receptor CXCR4 plays a central role in guiding cell migration along gradients of stromal cell-derived factor-1 (CXCL12). By genetically engineering dermal fibroblasts to overexpress CXCR4 prior to membrane harvesting, fibroblast membrane-coated nanoparticles with enhanced chemokine-guided homing capability can be obtained. In a recent study, CXCR4-engineered fibroblast membrane nanovesicles exhibited markedly improved accumulation in CXCL12-enriched tumor microenvironments through the CXCR4/CXCL12 axis. This receptor-mediated targeting significantly enhanced therapeutic efficacy, confirming that genetic programming of fibroblast membranes represents an effective strategy for chemokine-directed delivery using cell membrane-coated nanocarriers [111].

### 2.3. Fabrication Process for Biomimetic Membrane Materials

**Figure 2:**
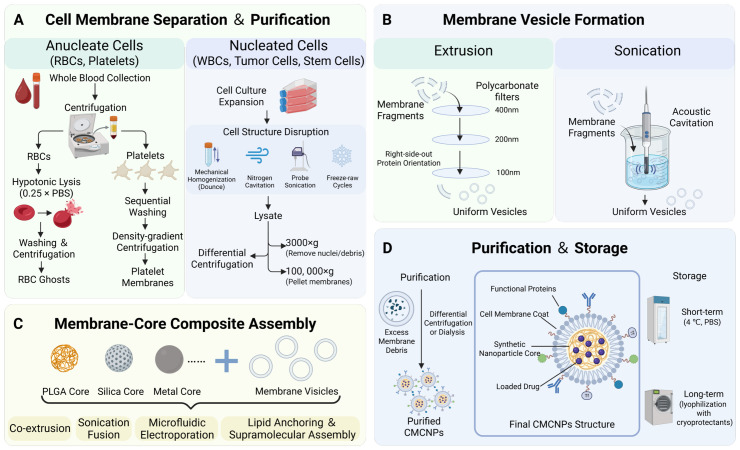
Fabrication process of CMCNPs: (**A**) cell membrane separation and purification, (**B**) membrane vesicle formation, (**C**) membrane-core composite assembly and (**D**) purification and storage. (Created in BioRender. Lin, Z. (2026). https://BioRender.com/j7q4rqf, accessed on 26 May 2026).

The manufacturing of biomimetic membrane for CMCNPs requires meticulous control over cell culture, membrane isolation, and coating efficiency to preserve the innate biological functions of the source cells. The fabrication process of CMCNPs are illustrated in Figure 2.

#### 2.3.1. Cell Membrane Separation and Purification

The fabrication begins with the harvesting and expansion of target cell populations in bioreactors or standardized cell culture systems. For anucleate cells, such as RBCs and platelets, the isolation process is relatively straightforward: whole blood is collected, and the specific cell fractions are separated via centrifugation [25]. RBCs typically undergo hypotonic lysis (e.g., 0.25× PBS), inducing osmotic swelling and subsequent rupture of the plasma membrane to release hemoglobin. The resulting “ghosts” are then washed and concentrated through centrifugation [112]. Platelet membranes are usually isolated by sequential washing and density-gradient centrifugation, to protect the membrane surface protein [113]. In contrast, nucleated cells—including macrophages, tumor cells, and stem cells—require more vigorous disruption techniques to effectively isolate the plasma membrane while removing the nucleus and organelles. Various specialized methods are employed depending on the type of cells and required membrane integrity, such as nitrogen cavitation for rapid and controlled rupture [114], mechanical homogenization (e.g., using a Dounce homogenizer or Polytron) to exert precise shear forces [24,115], gentle probe sonication to fragment membranes through acoustic cavitation [24,116], or hypotonic lysis combined with repeated freeze–thaw cycles to destabilize the cellular structure [24,115]. The lysate is subsequently purified through differential centrifugation (typically 3000× *g* to remove nuclei and intact cells, followed by 100,000× *g* to pellet plasma membrane fragments) and density-gradient separation to enrich membrane purity [24]. However, membrane isolation is not merely a separation process, as the preservation of membrane asymmetry, lipid integrity, and functional transmembrane proteins critically determines the biological performance of CMNPs. Excessive mechanical shear or prolonged sonication may disrupt membrane architecture and denature key surface proteins involved in immune evasion and cellular recognition. Conversely, insufficient disruption often results in contamination from intracellular organelles, cytoskeletal components, or nuclear debris. Therefore, isolation parameters—including osmotic strength, cavitation pressure, homogenization intensity, and centrifugation speed—must be carefully optimized according to cell type and membrane fragility [24,115].

#### 2.3.2. Membrane Vesicle Formation

To facilitate the coating process, purified membrane fragments must be transformed into uniform, nanoscale membrane vesicles. This is commonly achieved through two primary methods:

- Extrusion: The membrane suspension is repeatedly passed through polycarbonate membranes with defined pore sizes (e.g., 400 nm, 200 nm, and 100 nm) [24,25,46,115]. This mechanical shear force produces vesicles of consistent size and promotes a “right-side-out” orientation of membrane proteins, which is critical for downstream biological recognition [25].
- Sonication: Ultrasonic energy is applied to disperse membrane fragments into smaller vesicles [24,25,116]. While efficient and scalable, sonication parameters must be carefully optimized to prevent the denaturation of delicate surface proteins or inconsistent vesicle size distribution [25,116].

#### 2.3.3. Membrane-Core Composite Assembly

The fusion of membrane vesicles with synthetic nanoparticle cores, such as poly(lactic-co-glycolic acid) (PLGA), silica and metallic nanoparticles, represents the most critical assembly step. Several strategic approaches have been developed:

- Co-extrusion: The mixture of vesicles and nanoparticle cores is repeatedly extruded through small-pore membranes. This method is considered the gold standard for achieving high coating uniformity and structural integrity [19,20,24,117].
- Sonication Fusion: Brief ultrasonic treatment induces the spontaneous fusion of vesicles around the nanoparticle cores. Although simple and scalable, it may result in lack of uniformity [20,118].
- Microfluidic Electroporation: This advanced technique utilizes microfluidic devices where nanoparticles and vesicles are subjected to precise electric pulses [116,119]. The electric field creates transient aqueous pores in the lipid bilayer, allowing the core to enter the vesicle [120,121]. This method preserves membrane protein activity and recognition properties better than mechanical extrusion [116,119,120,121].
- Lipid Anchoring and Supramolecular Assembly: Innovative strategies have emerged to enhance coating stability. Lipid anchoring involves incorporating amphiphilic molecules, such as mono-acyl phosphatidylcholines (MPCs), to stabilize vesicles and promote robust fusion with cores [122]. Alternatively, supramolecular assembly utilizing host–guest interactions (e.g., β-cyclodextrin) allows for the nondestructive conjugation of membranes onto nanoparticles, preserving protein functionality that might be compromised by harsh mechanical forces [123].
- Electrostatic Self-Assembly and Direct Fusion: Spontaneous coating can occur if the nanoparticle core and membrane vesicles possess opposite surface charges [24,46]. In specific material combinations, membrane vesicles may also spontaneously fuse with cores through optimized co-incubation [115].

Despite the widespread adoption of extrusion and sonication strategies, membrane-core fusion efficiency remains highly dependent on multiple physicochemical parameters. The membrane-to-core ratio directly influences coating completeness, while nanoparticle size, curvature, and surface charge affect membrane wrapping behavior and colloidal stability. In general, moderately negative surface charges favor stable membrane adsorption and reduce nanoparticle aggregation, whereas highly cationic cores may destabilize the lipid bilayer and induce membrane rupture [124]. Extrusion-based approaches typically provide more homogeneous coating and correct membrane orientation because repeated passage through nanoscale pores facilitates membrane reconstruction around the nanoparticle core under controlled mechanical stress. However, repeated extrusion may reduce manufacturing throughput and potentially lead to partial protein loss due to cumulative shear forces [115]. In contrast, sonication-mediated fusion is operationally simple and scalable, but localized heat generation and cavitation effects may compromise protein conformation and produce heterogeneous membrane coverage [24].

#### 2.3.4. Purification and Storage:

The final CMNPs are purified from excess membrane debris using differential centrifugation or dialysis [24,46,115]. To maintain the stability of the lipid bilayer and the activity of surface proteins, the particles are typically stored at 4 °C in phosphate-buffered saline (PBS) supplemented with protease inhibitors [25,46,115]. For long-term preservation, lyophilization with appropriate cryoprotectants can be employed to maintain structural integrity [24,25,46].

#### 2.3.5. Scalability and Manufacturing Challenges

Although current fabrication strategies have demonstrated promising preclinical performance, the scalable manufacturing of CMNPs remains a major translational challenge. Conventional extrusion-based fabrication suffers from low throughput, membrane loss during repeated extrusion cycles, membrane filter clogging, and limited batch-to-batch reproducibility, all of which complicate large-scale production [115]. Moreover, the heterogeneity of source cell populations may further introduce variability in membrane composition and protein expression profiles, thereby affecting the consistency of biological performance. While sonication-based approaches offer improved operational simplicity and scalability, uncontrolled cavitation and localized thermal effects may impair membrane protein functionality and reduce coating uniformity [24].

To overcome these limitations, microfluidic-assisted assembly platforms have emerged as promising alternatives for continuous and highly controllable CMNP production. Microfluidic systems enable precise regulation of flow rate, mixing kinetics, electroporation intensity, and membrane-core interaction time, thereby improving coating reproducibility and reducing particle aggregation [20]. Importantly, these systems are more compatible with automated manufacturing and Good Manufacturing Practice (GMP)-oriented production workflows. Nevertheless, substantial challenges remain regarding large-scale membrane sourcing, long-term storage stability, sterilization, and standardized quality-control criteria for clinical translation.

### 2.4. Performance Evaluation Standards for Oral-Specific Biomimetic Materials

Due to the distinct physiological and pathological environment of the oral cavity, biomimetic membrane materials used for oral diseases require a comprehensive set of evaluation criteria that extend beyond traditional nanomedicine benchmarks. Currently, no such standard evaluation criteria exist, so we propose the following recommended data.

#### 2.4.1. Physicochemical and Structural Characterization

- Membrane Integrity and Morphology: Transmission Electron Microscopy (TEM) is the primary tool for verifying the successful formation of the core–shell architecture [25,46]. Additionally, Atomic Force Microscopy (AFM) is utilized to assess surface topography, providing quantitative data on bilayer uniformity and surface roughness at the nanoscale [25]. In the context of oral diseases, membrane integrity is critical for penetrating the dense extracellular polysaccharide (EPS) matrix of cariogenic and periodontitis-associated biofilms. Even 20 nm nanoparticles show limited diffusion into intraorally formed dental biofilms, indicating that the EPS matrix acts as a formidable barrier; compromised coatings risk premature drug leakage before traversing this barrier to reach the infection site [125].
- Protein Retention and Orientation: The preservation of the source cell’s “antigenic fingerprint” is crucial. SDS-PAGE is employed to determine the total protein profile, while Western blotting confirms the presence of specific functional markers (e.g., CD47 for immune evasion or CXCR4 for homing) [25,46,111]. Flow cytometry and immunogold labeling further ensure that these proteins maintain their native, outward-facing orientation required for biological interaction [25]. Beyond biological targeting, retained and correctly oriented membrane proteins, glycoproteins, glycolipids, and sialylated glycans form a glycocalyx-like hydrated interface that provides steric and hydration repulsion in protein-rich media. Thus, protein retention and outward-facing orientation are not only functional markers, but also key contributors to stability [126]. In the oral cavity, correctly oriented membrane proteins are essential to prevent aberrant macrophage polarization that drives chronic periodontitis. Macrophage membrane-coated nanoparticles have been shown to neutralize inflammatory factors by inhibiting M1 polarization and promoting M2 polarization, thereby restoring the inflammation–osteogenesis coupling and facilitating alveolar bone regeneration [54].
- Size and Polydispersity: The hydrodynamic diameter of biomimetic nanoparticles (typically 50–200 nm) is a decisive factor for tissue penetration and pharmacokinetics. According to the principles of nanoparticle design, particles within this range optimize the balance between avoiding renal filtration (<5 nm) and minimizing splenic/hepatic sequestration (>200 nm) [127]. Dynamic Light Scattering (DLS) is used to monitor size distribution; a low polydispersity index (PDI < 0.2) is indicative of a stable, uniform suspension essential for reproducible clinical outcomes [46,127]. Within the oral pathology context, the EPS-rich biofilm matrix functions as a size-selective diffusion barrier: purely polysaccharide-based matrices impose notable size-dependent subdiffusive dynamics on nanometer-sized objects, effectively acting as molecular sieves that restrict the mobility of particles exceeding the matrix mesh size [128]. Therefore, precise control of nanoparticle size is indispensable for ensuring therapeutic access to biofilm-protected pathogens in carious lesions and periodontal pockets.
- Surface Charge: A successful coating typically induces a shift in zeta potential toward the value of the source cell membrane (usually negative, between −10 and −30 mV) [25,46]. Surface charge critically influences protein adsorption; while highly cationic particles are rapidly cleared from circulation, neutral or slightly anionic surfaces exhibit significantly prolonged half-lives and enhanced stability in biological fluids [129]. In the oral environment, surface charge governs interactions with salivary mucins; cationic surfaces (e.g., chitosan) undergo electrostatic complexation with negatively charged mucins, promoting particle aggregation and immobilization on the salivary pellicle [130]. Zeta potential characterization is therefore indispensable for predicting intraoral colloidal stability and mucosal transport.
- Stability in Bio-relevant Media: Given the unique oral milieu, the structural stability of the coating must be validated in simulated saliva containing mucins, electrolytes, and enzymes (e.g., amylase, lysozyme) to ensure the system does not prematurely dissociate before reaching the target site [131]. Pathological oral conditions further amplify this requirement: cariogenic biofilms generate acidic microenvironments (pH < 5.0) that can destabilize uncoated nanoparticles. Naha et al. demonstrated that uncoated nanozymes precipitated within one hour in saliva, whereas dextran-coated formulations maintained colloidal stability under identical conditions—underscoring that coating stability in bio-relevant media is a mandatory, not optional, characterization for oral applications [132].

#### 2.4.2. Functional Performance and Environmental Adaptation

- Targeting and Biofilm Penetration: In vitro uptake studies quantify the specificity of the system toward target cells (e.g., OSCC cells or gingival fibroblasts) [111,133]. For infectious diseases, the ability to breach mature bacterial biofilms is evaluated via 3D confocal laser scanning microscopy (CLSM), measuring the depth of penetration (Z-stack) and the subsequent bacterial eradication rate [131].
- Mucoadhesion and Washout Resistance: A critical parameter for oral therapeutics is the resistance to salivary clearance. This is assessed using dynamic flow chamber models to simulate salivary flux [134,135]. The mechanical durability of the delivery system (e.g., oral thin films or hydrogels) is quantified via texture analysis, measuring the peak detachment force from mucosal tissues [134,136].
- Stimuli-Responsiveness: In diseased states like periodontitis, the evaluation focuses on the system’s sensitivity to acidic pH (5.0–6.0) or specific enzymes (e.g., gingipains), triggering controlled drug release at the site of inflammation [137,138].

#### 2.4.3. Biocompatibility and Safety Profile

The biological safety of oral biomimetic materials include: in vitro cytotoxicity (CCK-8/MTT assays) on oral cell lines [46]; hemolysis assays to ensure compatibility with inflamed, bleeding gingival tissues; and immunogenicity assessments to monitor pro-inflammatory cytokine release (e.g., TNF-α, IL-1β) from macrophages [139]. Long-term in vivo safety is further corroborated by histopathological and systemic biochemical analysis [122]. Note that thresholds such as IC50 values may vary depending on the specific material and cell type.

A comprehensive summary of key physicochemical and biological characterization indicators for oral biomimetic nanocarriers, together with corresponding detection methods and clinical significance, is presented in Table 2.

## 3. Applications of Biomimetic Cell Membrane Materials in Oral Diseases

**Figure 3:**
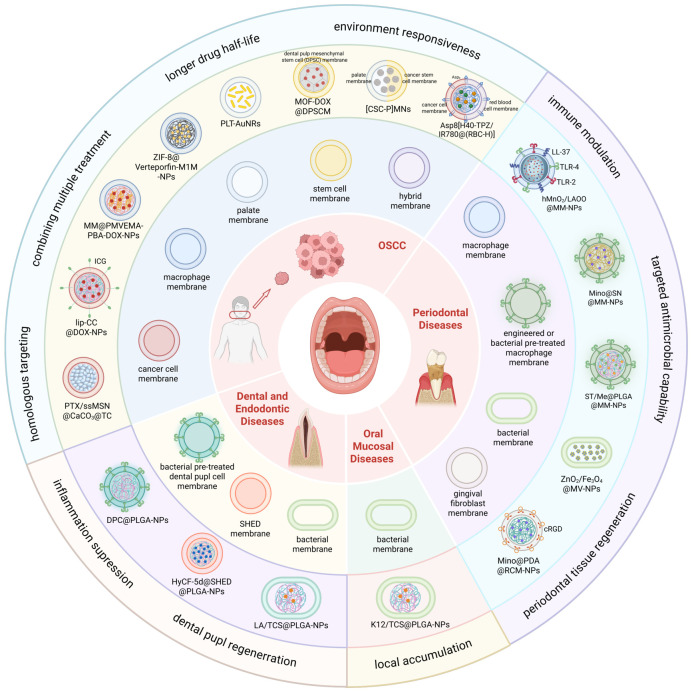
Applications of biomimetic cell membrane materials in oral diseases (Created in BioRender. Lin, Z. (2026). https://BioRender.com/xqzo567, accessed on 26 May 2026).

The following sections provide a detailed analysis of the application of biomimetic cell membrane-based drug delivery systems across four major categories of oral diseases, including oral squamous cell carcinoma, periodontal disease, dental and endodontic diseases, and oral mucosal disorders. An overview of these applications is schematically illustrated in Figure 3 (Applications of Biomimetic Cell Membrane Materials in Oral Diseases). Table 3 further summarized representative biomimetic nanoplatforms by membrane sources, fabrication strategies and distinct targeting mechanism for each oral disease.

### 3.1. OSCC

**Figure 4:**
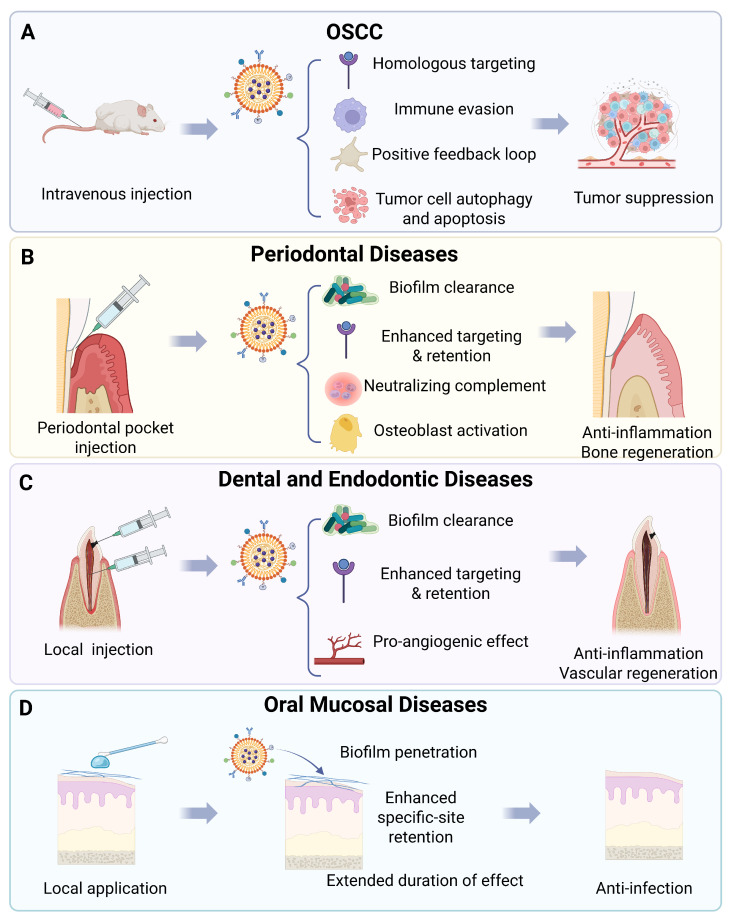
Common administration routes and mechanisms of action of CMCNPs in various oral diseases: (**A**) OSCC, (**B**) periodontal diseases, (**C**) dental and endodontic diseases and (**D**) oral mucosal diseases. (Created in BioRender. Lin, Z. (2026). https://BioRender.com/peo43x1, accessed on 26 May 2026).

OSCC is the most common type of oral cancers [141]. Despite advancements in conventional therapies such as surgery, radiotherapy, and chemotherapy, the overall prognosis for OSCC patients remains challenging, often due to drug resistance, systemic toxicity, and inadequate drug accumulation at tumor sites [141,142]. Biomimetic cell membrane-coated DDS, taking advantages of the unique properties of various cell membranes, including cancer cell membranes, represents enhanced targeting, prolonged circulation, and improved therapeutic efficacy (Figure 4A).

One of the most extensively explored biomimetic strategies for treating OSCC involves the use of CCMs for homotypic targeting. This approach capitalizes on the principle that CCMs retain specific surface proteins and receptors that facilitate recognition and adhesion to homologous cancer cells, thereby enhancing drug accumulation within the tumor microenvironment [143]. For instance, Liu et al. [144] developed paclitaxel (PTX)-loaded mesoporous silicon nanoparticles (MSNs) coated with Tca8113 cell membranes for homologous targeted therapy of tongue squamous cell carcinoma. These dual-responsive (pH/redox) nanoparticles, further coated with calcium carbonate, achieved a high PTX loading rate (9.68 ± 0.21%) and were administered via intravenous injection through the tail vein. They demonstrated excellent tumor-killing performance in vitro and in vivo. Quantitative in vitro assays demonstrated that 94.00 ± 1.66% and 98.12 ± 0.28% of Tca8113 cells were killed after 1 and 3 days of culture, respectively, with high homologous selectivity confirmed by negligible uptake in L929 and HeLa cells. The cancer cell membrane coating provided specific targeting capabilities, allowing the nanoparticles to home in on OSCC cells, which is a critical advantage over non-targeted systems. Similarly, Li et al. [145] engineered cancer cell membrane-camouflaged nanocarriers (CC@DOXNPs) for synergistic chemo/photothermal therapy of oral cancer, achieved through the synergistic action of indocyanine green (ICG) modified with the phospholipid polymer DSPE-PEG_2000_ introduced onto the HSC-3 cell membrane and doxorubicin (DOX) loaded within the polymeric core. In mice model, after administration via tail vein injection, the lip-CC@DOXNPs exhibited significant accumulation in tumors and effectively suppressed tumor growth under near-infrared (NIR, 808 nm) irradiation without systemic toxicity. RNA sequencing and gene expression analyses revealed that this combined therapy triggered mitochondria-mediated apoptosis via the p53 pathway, highlighting a multifaceted therapeutic mechanism. Wu et al. [146] designed CAL-27 cell membrane-coated mesoporous silica nanoparticles (MSNs) encapsulating Chlorin e_6_ (Ce_6_) and Curcumin (Cur) to amplify photodynamic therapy (PDT). Curcumin, a natural compound with anti-cancer activities [147], played a crucial role by disrupting the ROS-defense system through suppressing thioredoxin reductase (TrxR) activity and decreasing TrxR-2 expression, thereby significantly enhancing the cancer cell-killing ability of PDT. The biomimetic coating ensured selective accumulation in tumors, leading to substantial tumor growth inhibition in vivo. Exploring precise integration of PDT and autophagy inhibition in oral cancer, Dai et al. [148] used metal-organic framework material PCN-224 as a carrier for the autophagy inhibitor chloroquine (CQ) and coated with CAL-27 cell membrane, this system targeted tumor cells, enhancing retention in the tumor microenvironment. PDT activation generated ROS to induce apoptosis, while CQ inhibited autophagy, a common resistance mechanism in cancer cells, synergistically enhancing tumor suppression. There are many combinations of treatment methods. To combine photothermal therapy (PTT) and PDT with autophagy inhibition, Shi et al. [149] developed a biomimetic nanoparticle system using CAL-27 cell membrane vesicles to load photosensitizer ICG, a photothermal agent, and the autophagy inhibitor HCQ. These nanoparticles efficiently delivered drugs to oral tongue squamous cell carcinoma cells, and upon NIR laser (808 nm) irradiation, demonstrated significant antitumor efficacy both in vitro and in vivo (intravenous injection). This strategy addresses the challenge of autophagy induction by PTT/PDT, which can reduce therapeutic efficacy [149]. And Yin et al. [150] developed a cascade nanoreactor (δ-ALA@FMMSH-TPP-TCM) integrating chemodynamic therapy (CDT) and PDT for OSCC. Utilizing iron oxide and mesoporous silica, the FMMSH drug delivery system, encapsulating the photosensitizer prodrug δ-aminolevulinic acid (δ-ALA), is developed. Triphenylphosphine (TPP) modification facilitates mitochondrial targeting, while tumor cell membrane coating provides homotypic targeting. Esterase overexpression in the tumor triggered δ-ALA release, and Fenton chemistry in tumor mitochondria enhanced CDT. The synergistic PDT/CDT increased cytotoxic ROS levels, enhancing apoptosis. Sun et al. [151] combined PTT and radiotherapy using KB cell membrane-coated gold nanorods (GNR@Mem). GNR@Mem exhibited excellent photothermal and radiosensitizing properties, with the CCM coating providing stability and selective targeting. Under NIR light and X-ray irradiation, GNR@Mem induced temperature increase, reactive oxygen generation, and DNA damage, leading to apoptosis. The prolonged circulation time and homotypic targeting significantly increased tumor accumulation, resulting in suppressed tumor growth in vivo after 24 h post-intravenous-injection without systemic toxicity. Considering the heterogeneity, Wu et al. [152] created Au@C-CCM using CCMs from CAL-27 cell and patient-derived cells, represented optimal targeting properties for the highest therapeutic efficiency, suggesting a personalized medicine approach. The Au@C-CCM platform achieved a photothermal conversion efficiency of up to 44.2%. Additionally, to overcome the poor permeability of chemotherapy drugs into bone tissue, Chen et al. [153] designed bone-targeted erythrocyte–cancer hybrid membrane-camouflaged nanoparticles (Asp_8_[H40-TPZ/IR780@(RBC-H)]) for enhancing photothermal and hypoxia-activated chemotherapy of bone invasion. These nanoparticles were camouflaged with a head and neck squamous cell carcinoma WSU-HN6 cell (H) and RBC hybrid membrane, which were modified by an oligopeptide of eight aspartate acid (Asp_8_), exhibiting dual targeting (bone and cancer), immune escape properties from the erythrocyte membrane, and effective cancer growth inhibition.

Except cancer cell membranes, other cell membrane types have been explored for OSCC. The macrophage membrane provides a stealth effect and potentially active targeting to the inflammatory components within the tumor microenvironment, which is often rich in immune cells and inflammatory mediators [154]. Yang et al. [155] designed macrophage membrane-camouflaged pH-sensitive nanoparticles (MM@DOX NPs) for targeted therapy of OSCC. Administered via intravenous injection, MM@DOX NPs exploited macrophage membrane-mediated immune evasion to prolong systemic circulation. In vivo biodistribution confirmed enhanced accumulation at the tumor site. Pharmacokinetic analysis revealed a significantly extended half-life of 9.26 h for MM@DOX NPs compared with only 1.94 h for free DOX, and the pH-sensitive boronate ester bonds enabled selective DOX release at the acidic tumor microenvironment (pH 5.5). In HN6 tumor-bearing mice, MM@DOX NPs achieved significantly greater tumor suppression than free DOX with good biocompatibility. Yu et al. [156] utilizing macrophage membrane to coat Verteporfin-loaded zeolitic imidazolate framework 8 (ZIF-8), demonstrated efficient cellular uptake and apoptosis induction in CAL-27 cells upon laser irradiation. Coating these nanoparticles with macrophage cell membrane significantly enhanced circulation time and tumor-specific targeting in vivo (intravenous injection), leading to substantial tumor growth inhibition in nude mice.

Platelet membranes have also been utilized due to their natural ability to adhere to damaged endothelium and tumor vasculature, as well as their role in immune evasion [157]. Rao et al. [158] investigated platelet-facilitated photothermal therapy (PLT-PTT) for HNSCC, a broader category that includes OSCC. The Gold nanorods (AuNRs) loaded into platelets (PLT-AuNRs) inherited long blood circulation and cancer targeting characteristics from PLTs. In HNSCC-bearing Tgfbr1/Pten 2cKO mice model, after intravenous injection, PLT-AuNRs combined with laser irradiation effectively inhibited HNSCC growth. A notable finding was the self-reinforcing targeting mechanism: initial PTT-mediated tumor ablation induced local vascular damage that further recruited circulating PLT-AuNRs, amplifying therapeutic efficacy in a positive-feedback manner. Building on this, Bu et al. [159] developed a platelet–cancer stem cell hybrid membrane-coated iron oxide magnetic nanoparticle ([CSC-P]MN) for enhanced photothermal therapy of HNSCC. Cancer stem cells (CSCs) are believed to be the clonogenic core of tumors, driving growth and progression, and targeting them is a significant challenge [160]. [CSC-P]MN conferred immune evasion, active targeting, MRI capabilities, and photothermal therapy, combined the advantages of both cell types for improved targeting and therapeutic efficacy. Administered intravenously, the hybrid platelet-CSC membrane conferred both P-selectin-mediated adhesion to tumor vasculature and CSC-targeting capability. The iron oxide magnetic core provided MRI imaging functionality, enabling theranostic integration of diagnosis and therapy in a single platform.

Another innovative approach involves using stem cell membranes to target OSCC. Zhou et al. [161] modified metal-organic framework (MOF) nanoparticles with dental pulp mesenchymal stem cell (DPSC) membranes (MOF@DPSCM). This strategy was based on the observation that OSCC cells secrete chemokine CXCL8, which attracts DPSCs expressing CXCR2. MOF@DPSCM showed specificity for OSCC and were loaded with DOX. MOF-DOX@DPSCM effectively induced CAL27 cell death in vitro and blocked tumor growth in vivo (intravenous injection). The DPSC membrane provides a natural homing mechanism to the tumor, leveraging the tumor’s own signaling pathways. This approach is distinct from using cancer cell membranes for homotypic targeting, as it utilizes the migratory properties of stem cells towards the tumor microenvironment.

CMCNPs can also be combined with hydrogels to form composite drug delivery systems. Wu et al. [162] developed a physiologically responsive nanocomposite hydrogel for HNSCC treatment via proteolysis-targeting chimeras (PROTAC) enhanced immunotherapy. This hydrogel loaded imiquimod-encapsulated CaCO_3_ nanoparticles and cancer cell membrane-coated mesoporous silica nanoparticles containing PROTAC for BMI1 and paclitaxel. BMI1 has been found to promote the tumorigenesis, progression, and metastasis of multiple carcinomas/malignancies, including glioblastoma [163], prostate cancer [164], and HNSCC [165]. This nanocomposite hydrogel was designed for local peritumoral injection, forming a drug depot that degrades in response to tumor microenvironment (TME) acidity to sustainably release components, activate the T-cell immune response, degrade BMI1, and elicit apoptosis. The localized delivery strategy minimized systemic exposure of immunomodulatory agents, thereby reducing the risk of systemic immune-related adverse effects while achieving effective suppression of HNSCC growth and metastasis.

The above examples collectively highlight several key considerations for OSCC nanotherapy:

Upon systemic administration, serum proteins spontaneously adsorb onto the nanoparticle surface, forming a protein corona that redefines the particle’s biological identity and profoundly influences its pharmacokinetics, biodistribution, and tumor accumulation efficiency. Although CMCNPs show significantly reduced non-specific protein adsorption compared to bare nanoparticles, they still form a protein corona upon exposure to biological fluids [166]. However, the CMCNP protein corona is dominated by proteins that specifically interact with membrane proteins rather than the non-specific opsonins that characterize bare nanoparticle coronas [167]. This unique corona only minimally modulates CMCNP biological identity, preserving their homologous targeting and immunomodulatory functions. So the membrane coating integrity remains critical, as incomplete coating exposes the nanoparticle core and increases non-specific protein adsorption. Moreover, a deep understanding of the TME is required to optimize such interactions; a recent single-cell and spatial transcriptomic study revealed that the ANXA1-FPR2 signaling axis drives spatial communication between tumor cells and myeloid-derived suppressor cells (MDSCs), sustaining immune suppression and limiting therapeutic responses [168]. This finding highlights that effective biomimetic systems must not only navigate the protein corona but also actively disrupt these endogenous immunosuppressive pathways.

Targeting specificity is governed by retained membrane receptors: cancer cell membranes exploit homophilic adhesion molecules (E-cadherin, integrins) for homologous recognition; platelet membranes engage P-selectin/CD44 on tumor vasculature; macrophage membranes respond to chemokine gradients (CCL2/CCR2, CXCL12/CXCR4); and stem cell membranes utilize the CXCL8/CXCR2 axis. The CXCR4/CXCL12 signaling axis has been validated as a therapeutically relevant homing pathway, with CXCR4-engineered fibroblast membrane nanovesicles demonstrating chemokine-navigated tumor accumulation [111].

Many systems exploit the acidic TME (pH 5.5–6.8) via acid-labile bonds (boronate esters, hydrazones, calcium carbonate) or the elevated intracellular glutathione (2–10 mM) to cleave disulfide bonds, enabling site-specific drug liberation. Charge-reversal nanocarriers that switch from anionic/neutral to cationic upon TME triggers further improve tumor penetration. The integration of PTT, PDT, chemotherapy, radiotherapy, and immunotherapy within a single membrane-coated nanoplatform is a promising direction. The [CSC-P]MN platform combines MRI and PTT; PROTAC-loaded hydrogels merge chemotherapy with T-cell-mediated immunotherapy. To fully realize the potential of these combination immunotherapies, robust predictive biomarkers are needed. A recent study by Gong et al. established a cuproptosis-related lncRNA signature that effectively stratifies OSCC patient prognosis and predicts immunotherapy responses, offering a tool to guide personalized multimodal strategies [169].

While these strategies significantly improve tumor selectivity and therapeutic potency, several challenges remain. The translational relevance of homotypic targeting is limited by inter- and intra-patient tumor heterogeneity; a cell membrane derived from one cell line may not fully recapitulate the antigenic repertoire of a patient’s tumor. Additionally, the majority of studies rely on subcutaneous xenograft models that fail to replicate the complex oral microenvironment, including saliva, mechanical stress, and polymicrobial flora. Future work must validate these platforms in orthotopic, immune-competent models and address scalable membrane sourcing and quality control before clinical translation can be envisioned.

### 3.2. Periodontal Diseases

Periodontitis is a chronic inflammatory disease caused by the interaction of oral microorganisms with the host immune response. The pathogenesis of periodontitis involves complex interactions between pathogenic bacteria, host immune responses, and inflammatory mediators, often exacerbated by systemic conditions like osteoporosis [170]. Conventional treatments face challenges in effectively eradicating biofilms, modulating inflammation, and promoting tissue regeneration. CMCNPs offer a sophisticated approach to address these multifaceted challenges by providing targeted drug delivery, enhanced antibacterial activity, immunomodulation, and support for tissue repair (Figure 4B).

The macrophage membrane-coated nanoparticles delivering antimicrobial agents directly to the pathogen while simultaneously modulating the host immune response. Lin et al. [171] engineered macrophage membrane-camouflaged nanodecoys to reshape the infectious microenvironment for efficient periodontitis treatment. These nanodecoys were designed with recombinant LL-37 anchored to Toll-like receptor-enriched macrophage membranes, which not only eliminated bacteria but also ensured retention of the nanodecoys at the infection site. Furthermore, the incorporation of L-amino acid oxidase (LAAO) and hollowed manganese dioxide (hMnO_2_) accelerated oxygen generation and disrupted bacterial metabolism, contributing to enhanced antibacterial effects. This system was administered via local injection into the periodontal tissue. In vivo, the combined actions of LL-37-mediated bacterial elimination, LAAO/HMnO_2_-enhanced oxygen generation, and Nrf2 nuclear translocation resulted in reduced oxidative stress, promoted osteoblast differentiation, and significant alveolar bone regeneration as confirmed by histological and micro-CT analyses. Deng et al. [46] used modified macrophage cell membranes which expressing TLR4 and silk fibroin nanoparticles (SNs) loaded with minocycline hydrochloride (Mino) to prepare a biomimetic nanoparticle (MSNCs), for antibacterial and immunoregulatory dual-function treatment of ligature-induced periodontitis. They effectively targeted bacteria via TLR4, neutralized LPS—a major bacterial virulence factor that triggers inflammation [172]—and inhibited macrophage inflammation. In mouse model of ligature-induced periodontitis, locally injected into the periodontal pocket, MSNCs significantly reduced periodontal inflammation and alveolar bone loss, offering a promising new treatment strategy.

Another aspect of the biomimetic strategy involves bacterial pre-treated cell membranes and bacterial membranes, enabling the bacteria-targeting capability. *P. gingivalis*, one primary pathogen in periodontitis, has been reported to promote the progression of atherosclerosis [173]. Guo et al. [174] coated the PLGA–simvastatin–chitosan–metronidazole nanoparticles (STNPs) with *P. gingivalis*-treated macrophage membranes (MM/STNPs). These nanoparticles effectively eliminated *P. gingivalis* and converted macrophages from M1 to M2 phenotype. In mice model, after intravenous injection, MM/STNPs exhibited dual-site targeting capability, accumulating in both atheromatous plaques and inflamed periodontal tissue, reducing plaque formation and loss of alveolar bone. Similarly, Yan et al. [175] also developed a macrophage-based nanoformulation (MZ@PNM@GCP) for periodontitis treatment, using the *P. gingivalis*-LPS pretreated the macrophage membrane. This nanoformulation targeted *P. gingivalis* via Toll-like receptor complex 2/1 (TLR2/1), killing bacteria by disrupting their integrity and releasing metronidazole (MZ). It also prevented *P. gingivalis* from subverting the immune response and neutralized C5a, a potent inflammatory mediator. MZ@PNM@GCP restored immune function and killed bacteria with favorable biocompatibility in vitro and in vivo (periodontal pocket injection). Furthermore, *S. gordonii*, the initial colonizing bacterium [176], supports *P. gingivalis* to form symbiotic biofilms on gingival tissues with enhanced antibiotic resistance [177]. Cao et al. [178] reported a new strategy to treat periodontitis biofilms with *S. gordonii* membrane-coated H_2_O_2_ self-supplied nanocomposites (ZnO_2_/Fe_3_O_4_@MV NPs). On one hand, it achieves targeted action against *Streptococcus gordonii*. On the other hand, it utilizes active hydroxyl radicals (·OH) generated by ZnO_2_–Fe_3_O_4_@MV nanoparticles to eliminate the symbiotic biofilm formed by *Streptococcus gordonii* and *Porphyromonas gingivalis* through a hydrogen peroxide self-sustaining, nanocatalyst-assisted mechanism.

Other cell types and modifications also have been investigated. Pan et al. [179] developed a gingival fibroblast membrane-coated nanoparticle (PM@RCM), composed of minocycline-loaded polydopamine nanoparticles (PM) and cRGD-modified cell membranes (RCM). In vitro, PM@RCM rescued human periodontal ligament stem cells with antioxidant, anti-inflammatory, and pro-osteogenic effects. In vivo, after locally treatment, it promoted periodontal tissue regeneration in mice. And Wang et al. [180] demonstrated that membrane vesicles from MC-3T3 cells, a type of osteoblast, with enriched CXCR4 exhibited enhanced targeted delivery as drug carriers to inflammatory sites. Curcumin was encapsulated into CXCR4-CMVs (CXCR4/Cur-CMVs). These nanoparticles induced M2 macrophage polarization, exhibited anti-inflammatory effects, and improved homing via the CXCR4/CXCL12 axis in vitro. In vivo, CXCR4/Cur-CMVs aggregated within inflammatory areas after intravenous administration, significantly improving ulcerative colitis and apical periodontitis.

These studies highlight several critical design considerations for periodontitis. The localized, accessible nature of periodontal pockets justifies the prevalent use of intra-pocket injection, which minimizes systemic exposure. However, this route also demands high retention and sustained release within a flushed environment. Efficacy assessment requires multidimensional endpoints—micro-CT bone volume, histomorphometry, and cytokine levels—to capture both antimicrobial and immunomodulatory effects. Periodontitis-associated biofilms are polymicrobial communities with synergistic metabolic interactions that confer antibiotic tolerance through quorum sensing. An often overlooked dimension is fungal–bacterial synergy; conventional antibacterial-only strategies may inadvertently promote fungal overgrowth. A paradigm-shifting approach uses pH-responsive copper–gallic acid core–shell nanoplatforms (CGC@HSAF NP) that disrupt cross-kingdom synergy: the antifungal HSAF fractures biofilm scaffolds while Cu^2+^ kills bacteria and gallic acid scavenges ROS to reprogram the microenvironment [181]. Such ecological network disruption is more effective than single-species targeting.

Macrophage membrane-coated nanoparticles inherently retain TLR2/1 and TLR4 complexes that recognize PAMPs from *P. gingivalis*, simultaneously achieving bacterial targeting and LPS neutralization. Chemokine receptors (CCR2, CXCR4) retained on donor membranes respond to elevated CCL2/CXCL12 gradients in inflamed tissues, facilitating active recruitment. The mildly acidic periodontal pocket (pH 6.0–6.8) and elevated ROS provide exploitable triggers. An injectable pH-responsive hydrogel (MH@ZIF-8/CS/β-GP) undergoes sol-gel transition and sustained drug release specifically under acidic conditions [182], and a ROS-responsive injectable hydrogel with boronate ester linkages enables on-demand co-delivery of minocycline and anti-inflammatory agents [183].

Despite promising preclinical outcomes, the clinical translation of biomimetic periodontitis therapies faces significant barriers. First, the polymicrobial and patient-specific nature of periodontitis biofilms means that targeting a single species (e.g., *P. gingivalis*) may be insufficient; broad-spectrum approaches must not disturb commensal flora. Second, most studies evaluate short-term outcomes (weeks) in rodent models, while periodontitis is a chronic, relapsing condition requiring long-term efficacy and safety data. Third, manufacturing membrane-coated nanoparticles at scale with batch-to-batch consistency in protein orientation and activity remains technically challenging. Future research should focus on elucidating the long-term immunomodulatory effects and developing standardized potency assays for membrane-coated products.

### 3.3. Dental and Endodontic Diseases

Dental caries, commonly known as tooth decay, is a highly prevalent infectious disease caused by acid-producing bacteria, primarily *Streptococcus mutans*, which form biofilms on tooth surfaces. These biofilms lead to demineralization of enamel and dentin, resulting in cavities. Endodontic diseases, such as pulpitis, involve inflammation or infection of the dental pulp, the soft tissue within the tooth, often stemming from deep caries or trauma. Conventional treatments for caries involve mechanical removal and restoration, while pulpitis often requires root canal treatment, which can lead to loss of pulp vitality. Biomimetic cell membrane materials offer innovative strategies for both preventing caries by targeting cariogenic biofilms and promoting pulp regeneration or treating pulp inflammation (Figure 4C).

For dental caries, the primary goal is to disrupt and eliminate cariogenic biofilms. *Lactobacillus* species are well-known probiotics with inhibitory effects against *Streptococcus mutans* and its biofilm formation [184]. Leveraging these natural antagonistic properties, Weng et al. [94] coated triclosan (TCS)-loaded PLGA nanoparticles (TCS@PLGA-NPs) with an envelope of *Lactobacillus* (LA/TCS@PLGA-NPs) for antibiotic delivery against cariogenic biofilm and dental caries. The LA/TCS@PLGA-NPs exhibited the ability to adhere to *S. mutans* and integrate into existing biofilms. In vitro experiments demonstrated significant inhibition of biofilm activity, biomass, and virulence gene expression of *S. mutans*. In vivo studies further showed that the local treatment of LA/TCS@PLGA-NPs had a long-lasting inhibitory effect on caries progression in animal models. The safety profile of these nanoparticles was also favorable, suggesting their potential as effective candidates for caries prevention. The key advantage of using *Lactobacillus* cell envelopes is their inherent affinity for bacterial biofilms and their biocompatibility, which allows for targeted delivery of antimicrobial agents directly to the site of infection, minimizing systemic exposure and potential side effects.

In early pulpitis, when bacteria reach the pulp, dental pulp cells (DPCs) can act as front-line troopers to detect bacterial LPS via TLR4 [185,186]. LPS bind to DPCs and activate intricate intracellular signaling pathways, which then produce a plethora of inflammatory cytokines and amplify the inflammatory response [187]. Sun et al. [188] placed DPCs in an *E. coli* LPS-stimulated environment to induce high expression of Toll-like receptor 4 (TLR4) antigens and led to inhibition of the expression and secretion of multiple cytokines by inactivation of some inflammation-related intracellular signaling pathways. Subsequently, their cell membranes were fused onto PLGA nanoparticles. These nanoparticles, which were administered into pulpitis tissue via local injection, acted as “sentinels,” detecting and binding the LPS and competing with DPCs to effectively inhibit the inflammatory process in the early stage. This study confirmed the feasibility of using fibroblast membranes to create nanoparticles capable of regulating inflammation, expanding the potential cell types for inflammation management in dental applications.

Another critical challenge in pulp diseases is the regeneration of vital dental pulp tissue. Currently, researchers preliminarily achieve pulp regeneration by applying DPSCs [189,190] and stem cells from human exfoliated deciduous teeth (SHED) [191]. Compared to DPSCs, SHED exhibits faster proliferation and greater angiogenic capacity [191]. Chen et al. [192] found that the conditioned factor (CF) that SHED released under hypoxic condition (hyCF) showed a vascularizing effect. They invented the bionic dental pulp stem cells, encapsulating hyCF-5d into PLGA microspheres, which are subsequently encapsulated with cell membranes derived from SHED. These bionic dental pulp stem cells were designed for local injection into the root canal system. They can release pro-angiogenic factors, crucial for vascularization and regeneration. In both hindlimb ischemia and pulp regeneration mouse models, the bionic dental pulp stem cells significantly enhanced retention to ischemia site, restored blood perfusion, and 7.6-fold higher vascular density. This nanoparticle delivery platform that mimics stem cell functions address the challenges of low survival rates, immune rejection, and imprecise drug delivery associated with traditional stem cell transplantation, offering a more efficient, safe, and precise approach for endodontic regenerative therapy.

The applications to dental and endodontic diseases demonstrate the importance of microenvironment-tailored design. Caries and pulpitis represent distinct pathological milieus. Cariogenic biofilms generate a acidic microenvironment driven by *S. mutans* metabolism, serving as an endogenous trigger for pH-responsive antimicrobial release. In pulpitis, LPS from invading bacteria binds TLR4 on dental pulp cells, activating NF-κB and MAPK pathways. The competitive LPS interception strategy using dental pulp cell membrane-coated “sentinel” nanoparticles represents a unique paradigm that sequesters the initiating trigger before receptor engagement, rather than blocking a single downstream mediator.

While the “sentinel” concept for early pulpitis is elegant, its clinical application is limited by the diagnostic challenge of identifying early, reversible pulpitis before irreversible tissue damage occurs. The bionic dental pulp stem cell platform (SHED membrane) achieves impressive vascular density, but the regulatory path for membrane-coated cell-mimetic products is undefined. Furthermore, the dynamic oral environment (salivary flow, mastication, pH fluctuations) poses a major challenge for topical retention; long-term residence under these conditions must be demonstrated in vivo. Future innovations should integrate diagnostic capabilities to enable timely intervention and combine remineralization agents with antibiofilm activity for comprehensive caries management.

### 3.4. Oral Mucosal Diseases

Oral mucosal disease encompass a wide range of conditions, including infections, inflammatory lesions, and autoimmune diseases. Among these, oral candidiasis is currently most common one, caused by *Candida albicans*. When *C. albicans* hyphae adhere to oral epithelial cells and form biofilms, the expression of virulence factors increases, while susceptibility to antimicrobials and phagocytosis decreases significantly [193]. And traditional drug treatment regimens have difficulty adhering to mucous membranes [194]. Therefore, strategies that inhibit *C. albicans* adherence and biofilm formation are highly desirable. *Streptococcus salivarius* K12 is a well-known probiotic bacterium that has demonstrated inhibitory effects against *C. albicans* growth and adherence in vitro, and protective effects in oral candidiasis models in vivo [195] (Figure 4D).

Considering the above, Ye et al. [196] developed a bionic adherent oral mucosal drug delivery system, encapsulating *S. salivarius* K12 membranes on triclosan-loaded PLGA nanoparticles (K12/TCS@PLGA-NPs). Designed for topical oral mucosal application, K12/TCS@PLGA-NPs inherited the properties of the *S. salivarius* K12 source cell membrane, bionically adhering to the oral mucosa and binding to *C. albicans* hyphae. In an oral candidiasis model demonstrated significantly reduced *C. albicans* colonization and biofilm formation, with K12/TCS@PLGA-NPs achieving higher drug accumulation at the infection site compared with uncoated TCS@PLGA-NPs. The enhanced therapeutic efficacy was attributed to the synergistic combination of probiotic membrane-mediated targeting and triclosan-mediated antimicrobial action. The biomimetic nanocomplexes were considered safe and effective, holding considerable therapeutic promise for oral mucosal infections including candidiasis.

This probiotic membrane-based strategy exemplifies the value of leveraging natural oral microbial ecology. Oral mucosal infections require topical formulations with mucoadhesive properties, resistance to salivary flow and enzymatic degradation, and the ability to penetrate the mucus layer. The K12/TCS@PLGA-NPs system is rooted in competitive exclusion between commensal *S. salivarius* K12 and pathogenic *C. albicans*. The probiotic coating confers dual functions: specific adhesion to *C. albicans* hyphae via bacterial surface adhesins and enhanced mucosal retention, directly addressing the challenge of poor drug adherence.

The primary limitation of the application for oral mucosal diseases is its narrow spectrum; it targets only *C. albicans* and does not address mixed oral infections. Moreover, long-term treatment of probiotic membrane-coated particles requires thorough evaluation of potential immune sensitization or microbiome perturbation. The scalability of isolating and coating bacterial membranes from probiotic strains under GMP conditions is also unexplored. Future studies should explore the use of multi-species probiotic membranes or engineered hybrid membranes to broaden activity, and incorporate smart stimuli-responsive materials that sense and respond to multiple intraoral signals for on-demand therapy.

**Table 3:** Summary of biomimetic cell membrane-coated nanoplatforms for oral diseases.

Oral Disease & Application	Membrane Source	Fabrication Strategy	Targeting Mechanism	Ref.
OSCC–targeted chemotherapy	Tca8113 cancer cell membrane	PTX-loaded MSN with CaCO_3_ shell (pH/redox dual-responsive)	Homotypic targeting	[144]
OSCC–chemo/photothermal therapy	HSC-3 cancer cell membrane	Polymeric core loaded with DOX; DSPE-PEG-ICG inserted on membrane	Homotypic targeting	[145]
OSCC–amplified PDT	CAL-27 cancer cell membrane	MSN loaded with Ce6 and curcumin (redox disruption)	Homotypic targeting	[146]
OSCC–PDT + autophagy inhibition	CAL-27 cancer cell membrane	PCN-224 MOF loaded with chloroquine (CQ)	Homotypic targeting	[148]
OSCC–PTT/PDT + autophagy inhibition	CAL-27 cancer cell membrane vesicles	Co-loaded with ICG and hydroxychloroquine (HCQ)	Homotypic targeting	[149]
OSCC–cascade CDT/PDT	HN6 tumor cell membrane	Fe-MSN loaded with δ-ALA; TPP mitochondrial modification	Homotypic + mitochondrial targeting	[150]
OSCC–combined PTT/radiotherapy	KB cancer cell membrane	Gold nanorods (GNRs)	Homotypic targeting	[151]
OSCC–personalized PTT	CAL-27/patient-derived cancer cell membrane	Au@C-CCM nanoparticles	Homotypic targeting	[152]
Bone-invasive OSCC–PTT + hypoxia-activated chemo	Erythrocyte-WSU-HN6 hybrid membrane with Asp_8_ peptide	H40 loaded with IR780 and tirapazamine (TPZ)	Dual bone/cancer targeting + immune evasion	[153]
OSCC–targeted chemotherapy	Macrophage membrane	pH-sensitive NPs with DOX (boronate ester bond)	Macrophage-mediated immune evasion, inflammatory tropism	[155]
OSCC–targeted PDT	Macrophage membrane	ZIF-8 loaded with Verteporfin	Prolonged circulation, tumor tropism	[156]
OSCC–PTT	Platelet membrane	Platelets loaded with AuNRs	Platelet-mediated vascular targeting (self-amplifying)	[158]
OSCC–theranostic PTT/MRI	Platelet–cancer stem cell hybrid membrane	Iron oxide magnetic nanoparticles	P-selectin adhesion + CSC targeting, MRI	[159]
OSCC–targeted chemotherapy	Dental pulp stem cell (DPSC) membrane	MOF loaded with DOX	CXCL8/CXCR2 chemotaxis	[161]
OSCC–PROTAC-enhanced immunotherapy	Cancer cell membrane (in hydrogel)	Hydrogel + CCM-coated MSN loaded with PROTAC & PTX, plus imiquimod-CaCO_3_ NPs	TME-responsive, homotypic targeting	[162]
Periodontitis–antibacterial, immunomodulation, bone regeneration	Macrophage membrane with anchored LL-37	Nanodecoys loaded with LAAO and hMnO_2_	TLR-enriched membrane, bacterial recognition	[171]
Periodontitis–antibacterial, immunoregulation	Macrophage membrane (TLR4^+^)	Silk fibroin NPs loaded with minocycline	TLR4-mediated bacteria targeting, LPS neutralization	[46]
Periodontitis with atherosclerosis–dual-site therapy	*P. gingivalis*-pretreated macrophage membrane	PLGA–simvastatin–chitosan–metronidazole NPs	Dual-site (plaque, periodontium), M2 polarization	[174]
Periodontitis–antibacterial, immune restoration	*P. gingivalis*-LPS-pretreated macrophage membrane	Nanoformulation with metronidazole, C5a neutralization	TLR2/1 targeting of *P. gingivalis*	[175]
Periodontitis–biofilm eradication	*S. gordonii* membrane	ZnO_2_/Fe_3_O_4_ nanocomposites (H_2_O_2_ self-supply, ·OH generation)	Targeting *S. gordonii*, disrupting symbiotic biofilm	[178]
Periodontal tissue regeneration	Gingival fibroblast membrane (cRGD-modified)	Minocycline-loaded polydopamine NPs	cRGD targeting	[179]
Apical periodontitis–anti-inflammatory, homing	MC-3T3 osteoblast membrane vesicles (CXCR4-enriched)	Curcumin-loaded	CXCR4/CXCL12 axis, M2 polarization	[180]
Dental caries–anti-cariogenic biofilm	*Lactobacillus* membrane	PLGA NPs loaded with triclosan (TCS)	Affinity to *S. mutans* and biofilm	[94]
Early pulpitis–anti-inflammation	DPC membrane (LPS-stimulated, TLR4 high)	PLGA NPs	Competitive LPS interception	[188]
Pulp regeneration–pro-angiogenic	SHED membrane	PLGA microspheres with hypoxic conditioned factors (hyCF)	Stem cell membrane-mediated homing	[192]
Oral candidiasis–anti-fungal biofilm, mucosal retention	*S. salivarius* K12 membrane	PLGA NPs loaded with triclosan (TCS)	Adhesion to *C. albicans* hyphae, mucosal adhesion	[196]

## 4. Discussion and Outlook

Oral diseases, ranging from chronic inflammatory conditions such as periodontitis to aggressive malignancies like OSCC, remain a major clinical burden. Biomimetic cell membrane-based DDS have emerged as a promising solution. By integrating the intrinsic biological functionalities of natural cell membranes with engineered nanocarriers, these systems exhibit enhanced biocompatibility, immune evasion, prolonged circulation, and disease-specific targeting. Despite encouraging progress, several grand challenges remain that hinder clinical translation, necessitating a critical assessment of current strategies before their full potential can be realized.

The biological identity and long-term fate of biomimetic nanoparticles remain incompletely understood. Upon administration, the formation of a protein corona can mask targeting ligands and trigger recognition by the MPS, negating the immune-evasive properties of the membrane coating [197]. Beyond MPS clearance, a critical biosafety concern is the activation of the complement system, which can lead to complement activation-related pseudoallergy and further opsonization [198]. Furthermore, repeated administration raises the risk of immunogenicity. Allogeneic membrane sources can elicit anti-membrane antibodies, leading to the ABC phenomenon, which dramatically reduces the efficacy of subsequent doses and may cause hypersensitivity reactions [25]. These long-term biological interactions must be systematically profiled, especially for chronic oral conditions requiring repeated treatment. The variability arising from different cell sources, culture conditions, and patient-specific factors creates batch-to-batch inconsistencies in membrane protein composition and orientation, fundamentally affecting therapeutic reproducibility [25].

The path to the clinic is obstructed by significant manufacturing and regulatory obstacles. A primary technical hurdle is the lack of a standardized, scalable, and sterile manufacturing process [199]. Traditional membrane coating methods, such as co-extrusion, are labor-intensive and difficult to adapt to large-scale GMP production. While advanced microfluidic approaches like flash nanocomplexation offer a promising route toward scalable and homogeneous production [200], they are not yet universally adopted. Another critical bottleneck is terminal sterilization, a mandatory step for any injectable product. Conventional methods like autoclaving or gamma irradiation can degrade heat- or radiation-sensitive biological membranes, disrupting their structural and functional integrity; sterile filtration, on the other hand, is often incompatible with larger particles, presenting a significant dilemma [201]. From a regulatory standpoint, CMCNPs represent a paradigm shift that existing frameworks struggle to address. As products combining biological and nanomaterial components, they face an expanded evaluation scope that must encompass immunological safety, infectious safety (especially for human-derived materials), and rigorous raw material traceability. The lack of clear regulatory guidelines for such hybrid products creates uncertainty and impedes clinical development [202].

In our opinion, the route toward market availability will be stepwise. Within the next 3–5 years, the most realistic progress will likely be early human feasibility studies or point-of-care-oriented proof-of-concept studies, particularly for locally administered oral formulations (e.g., periodontal pocket delivery). These indications may be more clinically tractable than systemic delivery because local administration can reduce systemic exposure while allowing direct monitoring of therapeutic response and adverse reaction. In a 5–8 year horizon, routine public or commercial availability may become achievable for standardized, off-the-shelf formulations based on well-characterized allogeneic or stable cell-line-derived membranes, provided that scalable manufacturing, raw-material traceability, sterility assurance, and membrane-function assays are established. By contrast, systemic, autologous, or highly personalized CMCNP therapies for OSCC are likely to require a longer translational window, probably beyond 8–10 years, because they must additionally resolve donor-to-donor variability, individualized raw-material qualification, repeated-dose immunogenicity, and long-term biodistribution concerns.

Overcoming these barriers will require a multi-pronged effort. In the near term, developing standardized, robust quality control metrics—including cell membrane purity, protein activity, and batch-to-batch consistency—is paramount. The field must also embrace advanced bioprocessing technologies to establish a clear path to GMP-compliant, scalable production. Looking forward, the integration of stimuli-responsive design, multifunctional theranostic systems, and personalized therapeutic strategies is expected to further advance this field. Continued exploration of novel membrane sources, genetic engineering approaches to precisely tune membrane properties, and cross-disciplinary integration with regenerative medicine will likely open new avenues for precision treatment of oral diseases. Fostering early and proactive dialogue with regulatory agencies will be essential to align product development with clinical translation pathways.

## 5. Conclusions

In summary, biomimetic cell membrane-based systems represent a powerful and versatile platform in next-generation drug delivery. By bridging biological functionality with materials engineering, they hold significant potential to transform therapeutic strategies and improve clinical outcomes in oral disease management. However, as critically discussed, fully unlocking this potential demands concerted efforts to resolve fundamental challenges related to long-term biosafety and immunogenicity, manufacturing scalability, and regulatory science. Addressing these hurdles will be crucial for translating the remarkable preclinical promise of cell membrane-coated nanoparticles into tangible clinical benefits for patients with oral diseases.

## Figures and Tables

**Table 1:** Major source of biomimetic membrane: markers, targets, and advantages.

Membrane Source	Surface Markers	Biological Targets	Pharmacological & Physiological Advantages	Reference(s)
Erythrocyte	CD47, CD55, CD59, Spectrin, Actin	-	CD47–SIRPα “don’t eat me” signaling; spectrin–actin cytoskeleton for mechanical resilience and capillary deformation	[22,24,27,31,32]
Platelet	Integrins, P-selectin, GPIIb/IIIa, CD41	Tumor vasculature, *P. gingivalis*, *S. aureus*	Innate homing to vascular injury; site-specific adhesion to activated endothelium; pathogen-specific binding	[41,43,45,47,49]
Macrophage	TNF-αR, IL-6R, CD45	Pro-inflammatory cytokines (TNF-α, IL-6), OSCC cells	“Nanodecoy” cytokine sequestration; promotes M1-to-M2 phenotypic shift and alveolar bone regeneration	[46,53,54,56]
Neutrophil	$\beta_{2}$ integrins (LFA-1, Mac-1), PSGL-1	Inflamed endothelium (ICAM-1, P/E-selectins)	Natural leukocyte adhesion cascade recapitulation; targeted anti-inflammatory drug delivery	[57,58,59]
Dendritic/T/NK Cell	CCR7, LFA-1, CD45	Lymphatic system, activated endothelium	CCR7-directed lymphatic homing for OSCC metastasis; specialized immune modulation and guidance	[60,61,65,67]
Cancer Cell	CD44, E-cadherin, Galectin-3	Homotypic parent cancer cells (OSCCs)	“Cancer-catch-cancer” homotypic recognition; TAA-driven nanovaccine effects; penetration of biological barriers	[69,71,72,73,74]
Stem Cell (MSC)	CXCR4, CD44, CD147, CD138	SDF-1, PDGF, VEGF ligands	Chemotactic migration via “find me” signals; low MHC expression for immune evasion; intrinsic tissue-specific homing	[80,83,86,87]
Bacteria (OMV)	LPS, Porins, Flagellin	EPS-embedded biofilms, TLR4/5 receptors	High immunogenicity (PAMPs); Toll-like receptor activation; enhanced biofilm penetration in dental caries	[90,92,93,94,95]

**Table 2:** Physicochemical and biological evaluation standards for oral-specific biomimetic delivery systems.

Evaluation Category	Specific Parameter	Common Metric/Methodology	Clinical Relevance for Oral Health	Reference(s)
Structural Integrity	Core–Shell Architecture	TEM/AFM Visualization	Ensures cargo protection and biomimetic interface	[25]
	Protein Fingerprint	SDS-PAGE/Western Blot	Confirms retention of targeting and stealth markers	[25,111]
Physicochemical Properties	Particle Size & PDI	50–200 nm; PDI < 0.2 (DLS)	Optimizes mucosal penetration and avoids systemic clearance	[127]
	Surface Charge	−10 to −30 mV (Zeta Potential)	Contributes to colloidal stability and immune evasion	[25,129]
Delivery Function	Biofilm Penetration	CLSM	Critical for treating deep subgingival infections	[131]
	Targeting Effect	Fluorescence Imaging	Minimizes off-target effects in healthy oral mucosa	[133]
Oral Adaptation	Saliva Retention	Flow model	Prevents drug loss via involuntary swallowing	[134,140]
	Mucoadhesive Force	Detachment Force	Determines durability of local topical applications	[134,136]

## Data Availability

No new data were created or analyzed in this study. Data sharing is not applicable to this article.
